# Pandemic within a pandemic! Policy Implications of community-based Interventions to mitigate violence against women during COVID-19 in Urban Slums of Lucknow, India

**DOI:** 10.3934/publichealth.2023022

**Published:** 2023-04-27

**Authors:** Fnu Kajal, Ram Manohar Mishra, Amit Mehrotra, Vijay Kumar Chattu

**Affiliations:** 1 Department of Health Promotion Sciences, University of Arizona, Tucson, AZ 85719, USA; 2 UNICEF, India Country Office; 3 Department of Occupational Science & Occupational Therapy, Temerty Faculty of Medicine, University of Toronto, Toronto, ON, M5G 1V7, Canada; 4 Center for Transdisciplinary Research, Saveetha Dental College, Saveetha Institute of Medical and Technical Sciences, Saveetha University, Chennai- 600077; India; 5 Center for Evidence-Based Strategies, Global Health Research and Innovations Canada Inc. (GHRIC), ON, Toronto, Canada

**Keywords:** COVID-19, gender-based violence, vulnerable women, urban slums, domestic violence, violence against women, India

## Abstract

**Background:**

The COVID-19 pandemic has brought an unprecedented adverse impact on women's health. Evidence from the literature suggests that violence against women has increased multifold. Gender-based violence in urban slums has worsened due to a lack of water and sanitation services, overcrowding, deteriorating conditions and a lack of institutional frameworks to address gender inequities.

**Methods:**

The SAMBHAV (Synchronized Action for Marginalized to Improve Behaviors and Vulnerabilities) initiative was launched between June 2020 to December 2020 by collaborating with the Uttar Pradesh state government, UNICEF and UNDP. The program intended to reach 6000 families in 30 UPS (Urban Poor settlements) of 13 city wards. These 30 UPS were divided into 5 clusters. The survey was conducted in 760 households, 397 taken from randomly selected 15 interventions and 363 households from 15 control UPS. This paper utilized data from a baseline assessment of gender and decision-making from a household survey conducted in the selected UPS during July 03–15, 2020. A sample size of 360 completed interviews was calculated for intervention and control areas to measure changes attributable to the SAMBHAV intervention in the behaviours and service utilization (pre- and post-intervention).

**Results:**

The data analysis showed a significant difference (p-value < 0.001) between respondents regarding women's freedom to move alone in the control and intervention area. It also reflected a significant difference between control and intervention areas as the respondents in the intervention area chose to work for the cause of gender-based violence.

**Conclusion:**

The SAMBHAV initiative brought an intersectional lens to gender issues. The community volunteers were trained to approach issues based on gender-based violence with the local public, and various conferences and meetings were organized to sensitize the community. The initiative's overall impact was that it built momentum around the issue of applying the concept of intersectionality for gender issues and building resilience in the community. There is still a need to bring multi-layered and more aggressive approaches to reduce the prevalence of gender-based violence in the community.

## 1. Introduction

India is a growing economy; however, urbanization is still fascinating as the rate at which the urban population grows brings many unforeseen challenges. The slums were earmarked for the first time by the Census of India in 2001 [1]. According to the Slum Area Improvement and Clearance Act, India, urban slums are defined as dwellings considered unfit for residence for various reasons such as overcrowding, dilapidated surroundings, etc. UN-HABITAT also emphasizes the absence of safe water and sanitation facilities and enough living space to define a slum area [2]. The census of 2011 further categorized urban slums as Notified, Recognized and Isolated slums [3]. As per the census definition, All notified areas in a town or city notified as “Slums” by State, Union territories Administration, or Local Government under any Act, including a “Slum Act” may be considered as Notified slums. All areas recognized as “Slum” by State, Union territories Administration or Local Government, Housing and Slum Boards, which may not have been formally notified as slums under any act, may be considered as Recognized slums. A compact area of at least 300 population or about 60–70 households of poorly built congested tenements in the unhygienic environment, usually with inadequate infrastructure and proper sanitary and drinking water facilities. Such areas may be considered Identified slums. Urban slums are islands of poor socio-economic status among thriving urban areas. However, the current COVID-19 pandemic has posed severe challenges, especially for the urban poor in the slums. Unemployment rises, and the poor and marginalized conditions are bound to deteriorate further with a dip in the economy.

The UN estimated a global rise of 15% in domestic violence cases during a quarantine period of 90 days. The major causes and contributing factors are social isolation, increased stress, substance abuse issues, lack of economic independence and shortage of access to public resources [4]. A review of 63 global studies conducted in 2015 (pre covid times) corroborates that unemployment, alcohol abuse and less intervention by the government in rural areas account for more gender-based violence [5]. However, the literature also suggests that socio-economic inequalities are closely related to intimate partner violence [6],[7]. Based on caste, religion and socio-economic status, women in India have always been subordinate in the gender hierarchy, and the current pandemic has only widened these inequalities [8]. Lack of decision-making further aggravates the situation. There is a dearth of literature regarding the prevalence of violence in the urban slums of India in the current study context. National Family and Health Survey (NFHS-4) 2016 reveals that approximately 30% of women in India over 15 years of age have experienced domestic violence [9]. UN Women's global database on violence against women suggests that the prevalence of lifetime violence against women in India is about 28.8% [10]. The studies conducted in the urban slums of Mumbai reported that approximately 21–36% of women suffered from domestic violence [11],[12].

Physical distancing has become a new normal, and one has to learn to live with it. The COVID-19 pandemic has increased the number of domestic violence cases in India [13] and brought a halt to the clinical services for the victims of domestic violence [14]. Overall, it has endangered women's health in various ways, such as damaged mental health, lack of access to contraceptives, lack of social support, etc [15],[16]. Such a scenario calls for adopting innovative measures to combat new challenges while adapting ways to cope.

The country-wide lockdown since 24 March 2020 that became necessary to contain the spread of the COVID-19 pandemic brought an unprecedented humanitarian crisis due to the unprepared exodus of a massive population group of migrants. Most migrants moved back to their residence states and resided in slums. Relief measures such as the Government of India's *Pradhan Mantri Garib Kalyan* Yojana (Prime Minister Welfare for Poor) Package of 22.37 billion USD provided relief to around 800 million poor people by early August [17]. However, such measures seem to have bypassed migrants because of their highly informal existence in urban areas. Lack of institutional preparedness, safety net programs and corruption disrupt vulnerable populations' survival. [18]. Moreover, a study from slums in south India reported high seroprevalence rates among women than men indicating that there had been a lack of access for women to testing in urban slums [19]. Also, unfortunately, the government has not recognized it as a problem or put in place any system to prevent or rehabilitate such victims.

All this calls for urgent action and innovative media to address this pertinent issue which can happen only through community participation. However, there is a dearth of literature on the rigorous evaluation of any service delivery program, which is a hurdle in replicating best practices in another setting [20]. The high-intensity interventions evaluated as successful must be shared for cross-sharing of information with other parts of the world. One such initiative to identify vulnerabilities in Urban Poor Settlements (UPS) of Lucknow, the capital city of Uttar Pradesh, India, one of the hotspots of COVID-19, was taken up as Synchronized Action for Marginalized to improve Behaviors and Vulnerabilities (SAMBHAV) by the local government of Uttar Pradesh along with UNICEF and UNDP. This intervention focused on eight critical areas, one of which was gender and decision-making. This paper summarizes the results of pre-and post-data collection and analysis on gender issues. We aim to bring more knowledge and experience on gender and decision-making by sharing the results and describing the emerging policy options for reducing risks and minimizing vulnerabilities while improving the coordination of key stakeholders.

## 2. Interventions description

**Figure 1.**
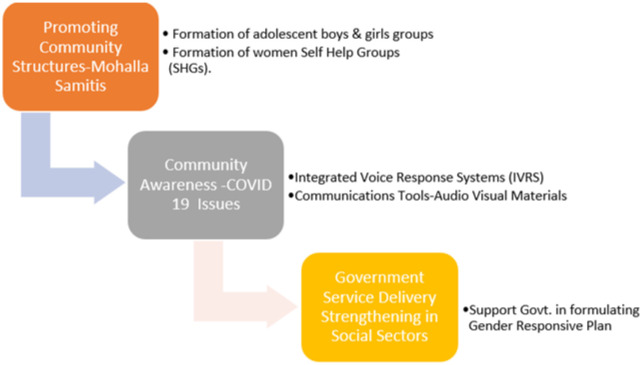
SAMBHAV interventions.

SAMBHAV is a pilot project implementing three interventions (Figure 1). The first set of interventions is promoting community structures, viz. *Mohalla Samitis*^1^, formation of adolescent boys & girls' groups and formation of women Self Help Groups (SHGs). These Mohalla Samitis represent people who are well respected within the community. The second set relates to providing knowledge to communities on key program behaviors using Integrated Voice Response Systems (IVRS), particularly during COVID-19 threat, and using various communication tools, viz. printed / audio-visual materials. The IVRS allowed us to push text messaging regularly to the participants through an available network provider. The third set of interventions is by strengthening Government service delivery on other critical social sectors, i.e., Water Sanitation and Hygiene, Child Health and Nutrition, Child Education, Child Protection, Promotion of livelihood and Social Protection Schemes among the most marginalized. For example, we strengthened the water and sanitation facilities by adding more community toilets, providing tablets for education sector etc. Thus, it allowed us to build better rapport with the community.

The first set of interventions is used to engage with the communities. The second set of interventions aims to improve knowledge and attitudes on various thematic issues. Components of the third set of interventions of the project's gender and decision-making activities were Capacitating community groups on Gender dimensions, i.e., providing training of Mohalla Samiti members on gender roles etc., Supporting the formulation of a Gender-responsive plan with the participation of community members, i.e. engaging the women of local slums in all activities that were being carried out in the slums during SAMBHAV intervention, and roll-out of its implementation.

- A handbook detailing social protection schemes and entitlements was developed, focusing on gender to help build awareness among UPS residents. A Mohalla Samiti (committee of ward members) was created to mobilize the community.
- Gender sensitization and training program was conducted for community-based organizations such as SHGs (Self-help Groups). Weekly interface meetings in the community in the presence of ward members and *Saajhi Duniya* played a crucial role in awareness building.
- Saajhi Duniya conducted an online distance training program, an expert group on gender, with all ward members (elected representatives from the slum community) to promote gender-responsive governance and transformative actions. This training also aimed to inform other training processes to be rolled out in all the identified city wards.

All these three sets of interventions are executed from mid–June 2020 to the end of December 2020. The first set of interventions was done during the first three months of SAMBHAV implementation when community facilitators of NGOs gradually worked and developed community platforms. The second set of interventions, i.e., IVRS messages, are sent to community members having mobile numbers- 16 times on various themes. The third intervention set is part of regular service delivery by Government service delivery platforms which is done mainly on a daily/ weekly basis.

***Background of Urban Slums in Lucknow:*** The last official enumeration of slums in Lucknow city was prepared for the Slum Free City Plan of Action between 2009 and 2013. It found 609 slums with a population of 148,117 households. Among them, 502 were said to be notified slums, while the remaining 107 were non-notified. Further, a survey was conducted by Vigyan Foundation in partnership with WaterAid, which identified 704 poor settlements in Lucknow as per the following:

**Table 1.** Type of settlements in Lucknow.

Type of settlement^2^	No	%
Urban Villages	245	35
Old colony/*Haata*	241	34
Unregistered slums	123	17
Slums registered under District Urban Development Agency (DUDA)	75	11
Rehabilitation Colony	20	3

***Demography:*** The estimated number of people living in these poor settlements is 294,912. The largest settlement mapped had approximately 8050 households. 150 was the most frequent number of households in a settlement, i.e., 54 settlements. Old colonies are concentrated in the old city of Lucknow, while urban villages are distributed throughout the municipal boundary. Registered slums are also concentrated in the old city, with some distributed in the south. As per survey responses, the oldest settlement was 700 years old, while the youngest was only 1 year old.

**Figure 2.**
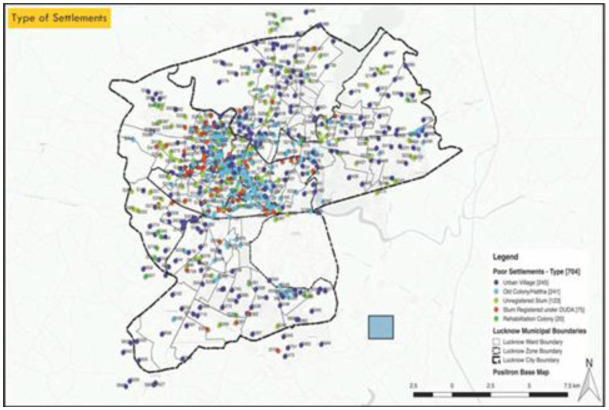
GIS Map of Lucknow showing the type of settlements.

## 3. Materials and methods

As per the census survey of 2011 Lucknow, with an approximate population of 2.8 million, the total number of slums is 65,629 [21]. The Figure 2 provides a snapshot of GIS mapping of type of settlements in Lucknow. The program intended to reach 6000 families in 30 UPS of 13 city wards. These 30 UPS were divided into 5 clusters. Each cluster established a support center for eight identified sectors: Water Sanitation & Hygiene, Livelihood, Social Protection, Health, Nutrition, Gender and decision-making, Education and digital divide and Child labor. The survey was conducted in 760 households, 397 taken from randomly selected 15 interventions, and 363 households from 15 control UPS. The control UPS were neighboring settlements as the UPS selected for intervention in terms of population size. This paper utilizes data from a baseline assessment, a household survey conducted in the selected UPS during July 03–15, 2020. The baseline assessment was part of a controlled, before-and-after trial to estimate the changes attributable to the SAMBHAV intervention. Before the intervention was applied, a baseline data collection was done. Since it was COVID time, we preferred to obtain verbal consent. The participants were explained about the government programs in both intervention and control areas. However, only the population in the intervention area was explained about the intervention. A sample size of 360 completed interviews was calculated for intervention and control areas to measure changes attributable to the *SAMBHAV* intervention in the behaviors and service utilization. These services related to household sanitation, hygiene, water safety, livelihood, child health and nutrition, education, child protection and social protection schemes.

All interviews were conducted by trained field staff from a reputed local non-government organization with substantial experience working with people living in UPS. All field staff had verbal and written skills in Hindi/ Urdu, the local languages of the survey localities. The survey instrument was developed by incorporating the standard questions (if available) used in Demographic Health Surveys (DHS) and Multiple Indicator Cluster Surveys (MICS) in English and translated into Hindi. The translated instrument was reviewed by study investigators fluent in both languages. Then there were pre-tested in communities similar to the survey sites. A customized interface was designed using Open Data Kit (ODK)^3^ technology for the data collection on Android mobile phones. The field staff was trained on using survey instruments using online training platforms.

The respondents were either the head of the household or adult household members. No names and addresses were recorded while collecting the information. Survey participants were not compensated but were provided information on measures to protect themselves from COVID-19 and information about social protection schemes.

## 4. Results

The demographic profile of the household studied in urban slums is given below in Table 2. The average household size in the study slums was 5.6 persons per household. About 44 per cent of the households follow Islam, and nearly 28 per cent belong to the scheduled castes/tribes, the most vulnerable section of Indian society. Almost 80 per cent of the households had only one member earning a livelihood. More than four-fifths (84%) of the households reported their average monthly income below ten thousand Indian Rupees. Membership in the self-help groups was dismal (about 1%).

**Table 2.** Demographic, social and economic characteristics of the households in intervention and control slums.

Characteristics	Total (N = 760)	Intervention (N = 397)	Control (N = 360)	P-value (significance of differences between intervention and control slums)
Average household size	5.6	5.6	5.6	0.847
Religion				
Islam	44.0%	38.3%	50.3%	0.001
Hindu	56.0%	61.7%	49.7%	
Caste				
SC/ST (Scheduled Caste/Schedules Tribes)	27.9%	33.5%	21.8%	<0.001
Others	72.1%	66.5%	78.2%	
Percent of households with only one person earning a livelihood	79.5%	81.6%	77.1%	0.127
Household monthly income (INR)				
Up to 10,000	84.7%	91.2%	77.7%	<0.001
10,001–15,000	9.2%	6.6%	12.1%	
More than 15,000	6.1%	2.3%	10.2%	
Percent of households with SHG membership	0.8%	1.5%	0.0%	0.019

**Table 3.** Women's autonomy and participation in household decisions.

Women's autonomy and participation in household decisions	Total (N = 431)	Intervention (N = 272)	Control (N = 159)	P-value for the significance of differences between intervention and control slums
Percent of currently married women (15–49 years) who usually make a decision by themselves about their health	11.6%	13.6%	8.2%	0.090
Percent of currently married women (15–49 years) who make decisions about major household purchases either by themselves or jointly with their husbands	72.2%	73.5%	69.8%	0.406
Percent of currently married women (15–49 years) who have access to money that they alone can decide how to use	47.1%	51.1%	40.3%	0.029
Percent of currently married women (15–49 years) who can go to the market alone	41.3%	41.2%	41.5%	0.946
Percent of currently married women (15–49 years) who can go to the health facility or medical store alone	39.9%	40.8%	38.4%	0.617

*Note: Asked only to those households with at least one currently married woman (15–49 years). If the household had more than one currently married woman in the age group (15–49) years, then one of them was randomly selected for the assessment.

Table 3 shows that there has been no significant difference in decision-making by women between intervention and control areas.

**Figure 3.**
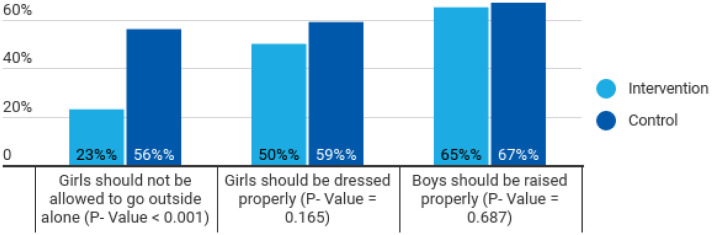
Comparison of responses between control and intervention groups to stop eve-teasing with girls.

**Figure 4.**
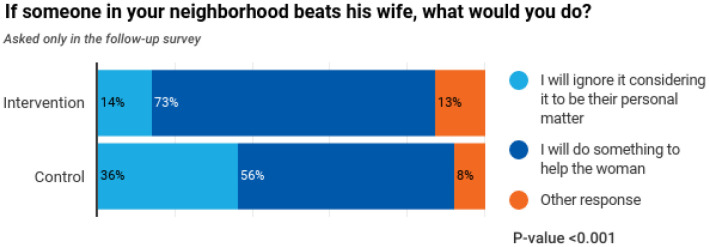
Response to wife-beating question in control and intervention.

Figures 3 and 4 are responses to the questions about gender violence and the reasons. Figure 3 shows a significant difference (p-value < 0.001) between respondents regarding women's freedom to move alone in the control and intervention area. This result might have arisen from the cautious behaviour that people would want to protect girls from an untoward incident. Figure 4 also reflects a significant difference between control and intervention areas as the respondents in the intervention area choose to work for the cause of gender-based violence. This shows that the three sets of interventions could significantly impact the community by sensitizing the community to the norms prevalent in the society that perpetrates gender-based violence.

## 5. Discussion

Violence against women has been continuing for ages. The COVID-19 pandemic has brought out its ugliness to the forefront. The state's health systems are weak, as there is no institutional way to prevent and care for the victims of violence. This pilot initiative has focused on interventions that mobilize the community and create ripple change from within the society. The above results indicate that the existing norms and attitudes are not mitigating gender-based violence. There is a need to transform the pre-existing norms and attitudes positively in a targeted manner. However, this alone is not enough. WHO established the RESPECT framework to design interventions at the policy level [22]. The strategies are- Relationship skills strengthening; Empowerment of women; Services ensured; Poverty reduced; Enabling environments (schools, workplaces, public spaces) created; Child and adolescent abuse prevented; and Transformed attitudes, beliefs and norms. The SAMBHAV intervention for preventing gender-based violence focused on ensuring service delivery and creating an enabling environment, along with some efforts to transform attitudes, beliefs and norms.

Studies have found that the lockdown enabled the perpetrators to engage in violence in the patriarchal society as the women had no option to escape or a lack of capable guardians [23],[24]. The containment strategy applied by the government did not understand the swing-like lifestyle of urban populations. The movement of mass exodus of informal migrants to the densely populated urban slums had put extra pressure on the existing resources. Lack of preparedness and necessary interdepartmental coordination structures only added to the crisis. Thus, the SAMBHAV initiative was launched to demonstrate the effectiveness of interdepartmental coordination and community mobilization for managing the COVID-19 pandemic. A similar study in Bangladesh has found that urban slums are marred by internal and external factors [18]. This initiative helped build resilience, which builds on society's transformative and adaptive nature.

While local-level interventions are necessary to ensure better service delivery, it is very pertinent to mention that these local interventions cannot work in isolation. The concept of intersectionality was introduced by Kimberle Crenshaw [25]. The SAMBHAV initiative incorporated this intersectionality lens to reduce the vulnerabilities of the urban slums, especially women. The other sets of interventions on livelihood generation targeted unemployment, thus, engaging perpetrators more productively and indirectly reducing gender-based violence in the community. The government must recognize that gender-based violence has to be addressed and, thus, create effective legislation during such exigencies.

The limitations of SAMBHAV have been recognized as it only lasted for about six months; thus, the authors believe that the impacts might be minuscule. However, it did start a momentum to be built around this issue. There is a need to replicate this initiative at various locations to understand the cultural differences that might be attributable to this issue. The authors recommend developing more elaborative research around this issue in the urban slums as it has neither caught the attention of researchers nor the government.

## 6. Conclusions

The authors conclude that there is a dearth of evidence of gender-based violence in the urban slums of India, especially in Uttar Pradesh, the country's most populous state. There is a need for multi-layered and more aggressive approaches to target this issue from various perspectives, such as masculinities. **The following recommendations are drawn from this pilot intervention:**

- Community resilience is required to combat gender-based violence.
- Ensure the effectiveness of the intervention by creating an enabling environment, i.e., mobilize resources within the community and reinforce synergies between various departments.
- Including women and their voices for global health governance by changing social norms is the key.

Initiatives such as SAMBHAV informed that gender intersects with multiple sectors, so integrating this issue with other social determinants of health is required. We hope initiatives like SAMBHAV will open a pathway to bring an imperative change in dealing with gender-based violence in other parts of the country.

## Footnotes

1 Mohalla samiti: It is a committee consisting of 13–15 persons in a specified Urban Poor Settlement, majority of them being women.

2 Mohall Samiti- It is a committee consisting of 13–15 persons in a specified Urban Poor Settlement, majority of them being women. 5 Haatas are old compounds generally belonging to private/individuals where small rooms are rented out at nominal rates to families. In this classification ‘unregistered slums' are impermanent and/or poorly maintained dwellings with poor living conditions which have not been recognized by the government for improvement, while registered slums are those that have been identified by DUDA as slums. Urban villages are erstwhile villages that have slowly been included in municipal limits over the years.

3 Brunette W, Sundt M, Dell N, Chaudhri Rohit, Breit N, Borriello G (2013) Open data kit 2.0: expanding and refining information services for developing regions. Department of Computer Science and Engineering, University of Washington. Web: https://homes.cs.washington.edu/~anderson/papers/ICTD/ODK2_Hotmobile.pdf.

## References

[1] Ministry of Home Affairs (2011). Census of India Website: Office of the Registrar General & Census Commissioner, India.

[2] UN Habitat, Slums: Some Definitions, State of the World's Cities.

[3] Ministry of Home Affairs G of I (2011). Census of India Website: SRS Statistical Report.

[4] Bailey RK (2021). Intimate Partner Violence: An Evidence-Based Approach.

[5] Edwards KM (2015). Intimate Partner Violence and the Rural–Urban–Suburban Divide: Myth or Reality? A Critical Review of the Literature. Trauma Violence Abuse.

[6] Yakubovich AR, Heron J, Feder G (2020). Long-term Exposure to Neighborhood Deprivation and Intimate Partner Violence among Women: A UK Birth Cohort Study. Epidemiology.

[7] Jesmin SS (2017). Social Determinants of Married Women's Attitudinal Acceptance of Intimate Partner Violence. J Interpers Violence.

[8] Chakraborty D (2021). The “living dead” within “death-worlds”: Gender crisis and covid-19 in India. Gend Work Organ.

[9] International Institute for Population Sciences (2017). National Family Health Survey (NFHS-4) 2015–16 India.

[10] UN Women (2017). Violence Against Women and Women's Economic Empowerment.

[11] Gaikwad V, Rao DH (2014). A cross-sectional study of domestic violence perpetrated by intimate partner against married women in the reproductive age group in an urban slum area in Mumbai. Indian J Public Heal Res Dev.

[12] Begum S, Donta B, Nair S (2015). Socio-demographic factors associated with domestic violence in urban slums, Mumbai, Maharashtra, India. Indian J Med Res.

[13] Maji S, Bansod S, Singh T (2021). Domestic violence during COVID-19 pandemic: The case for Indian women. J Community Appl Soc Psychol.

[14] Johnson K, Green L, Volpellier M (2020). The impact of COVID-19 on services for people affected by sexual and gender-based violence. Int J Gynecol Obstet.

[15] Sharma P, Sharma S, Singh N (2020). COVID-19: Endangering women's mental and reproductive health. Indian J Public Health.

[16] Usher K, Bradbury Jones C, Bhullar N (2021). COVID-19 and family violence: Is this a perfect storm?. Int J Ment Health Nurs.

[17] Government of India D portal (2021). Pradhan Mantri Garib Kalyan Package (PMGKP) | National Portal of India.

[18] Surma SA, Hakim SS, Rahman Lushan MS (2021). Planning for Pandemic Resilience: COVID-19 experience from urban slums in Khulna, Bangladesh. J Urban Manag.

[19] George CE, Inbaraj LR (2021). Re: George et al. High seroprevalence of COVID-19 infection in a large slum in South India; what does it tell us about managing a pandemic and beyond?. Epidemiol Infect.

[20] WHO (2011). Responding to intimate partner violence and sexual violence against women.

[21] IndiaCensus.net, Lucknow Population 2021.

[22] World Health Organization (WHO) (2021). Violence against women.

[23] Krishnakumar A, Verma S (2021). Understanding domestic violence in India during COVID-19: a routine activity approach. Asian J Criminol.

[24] Onditi F, Obimbo M, Muchina SK (2020). Modeling a pandemic (COVID-19) management strategy for urban slums using social geometry framework. Eur J Dev Res.

[25] Ryan NE, El Ayadi AM (2020). A call for a gender-responsive, intersectional approach to address COVID-19. Glob Public Health.

